# O Treinamento Resistido Atenua a Remodelação Cardíaca Induzida por uma Dieta Hiperlipídica em Roedores: Uma Revisão Sistemática

**DOI:** 10.36660/abc.20230490

**Published:** 2024-04-11

**Authors:** Alexandre Martins Oliveira Portes, Sebastião Felipe Ferreira Costa, Luciano Bernardes Leite, Victor Neiva Lavorato, Denise Coutinho de Miranda, Anselmo Gomes de Moura, Leôncio Lopes Soares, Mauro César Isoldi, Antônio José Natali

**Affiliations:** 1 Universidade Federal de Viçosa Viçosa MG Brasil Universidade Federal de Viçosa, Viçosa, MG – Brasil; 2 Universidade Federal de Ouro Preto Ouro Preto MG Brasil Universidade Federal de Ouro Preto – Campus Morro do Cruzeiro, Ouro Preto, MG – Brasil

**Keywords:** Miócitos Cardíacos, Dieta Hiperlipídica, Treinamento de Força, Revisão Sistemática

## Abstract

**Fundamento:**

A obesidade está associada ao desenvolvimento de doenças cardiovasculares e constitui um grave problema de saúde pública. Em modelos animais, a alimentação com uma dieta hiperlipídica (DH) compromete a estrutura e a função cardíaca e promove estresse oxidativo e apoptose. O treinamento resistido (TR), entretanto, tem sido recomendado como coadjuvante no tratamento de doenças cardiometabólicas, incluindo a obesidade, porque aumenta o gasto energético e estimula a lipólise.

**Objetivo:**

Na presente revisão sistemática, nosso objetivo foi avaliar os benefícios do TR no coração de ratos e camundongos alimentados com DH.

**Métodos:**

Foram identificados estudos originais por meio de busca nas bases de dados PubMed, Scopus e Embase de dezembro de 2007 a dezembro de 2022. O presente estudo foi conduzido de acordo com os critérios estabelecidos pelo PRISMA e registrado no PROSPERO (CRD42022369217). O risco de viés e a qualidade metodológica foram avaliados pelo SYRCLE e CAMARADES, respectivamente. Os estudos elegíveis incluíram artigos originais publicados em inglês que avaliaram desfechos cardíacos em roedores submetidos a mais de 4 semanas de TR e controlados por um grupo controle sedentário alimentado com DH (n = 5).

**Resultados:**

Os resultados mostraram que o TR atenua o estresse oxidativo cardíaco, a inflamação e o estresse do retículo endoplasmático. Também modifica a atividade de marcadores de remodelamento estrutural, apesar de não alterar parâmetros biométricos, parâmetros histomorfométricos ou a função contrátil dos cardiomiócitos.

**Conclusão:**

Nossos resultados indicam que o TR parcialmente neutraliza o remodelamento cardíaco adverso induzido pela DH, aumentando a atividade dos marcadores de remodelamento estrutural; elevando a biogênese mitocondrial; reduzindo o estresse oxidativo, marcadores inflamatórios e estresse do retículo endoplasmático; e melhorando os parâmetros hemodinâmicos, antropométricos e metabólicos.

## Graphical abstract

**Figura Central:**
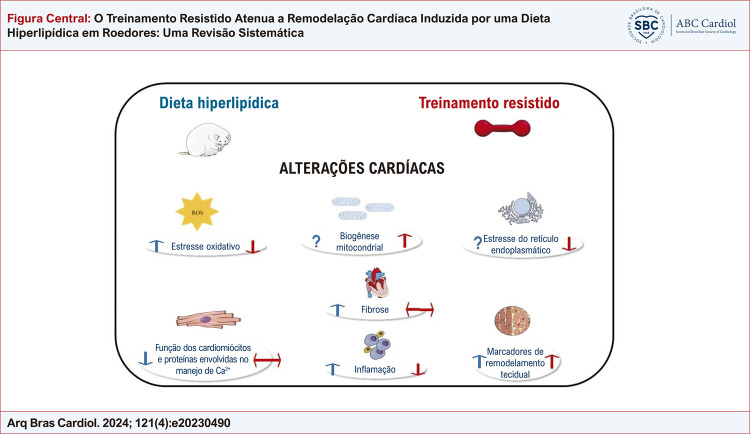
: O Treinamento Resistido Atenua a Remodelação Cardíaca Induzida por uma Dieta Hiperlipídica em Roedores: Uma Revisão Sistemática

## Introdução

Atualmente, a obesidade é um grave problema de saúde pública. Em 2016, mais de 1,9 mil bilhões de adultos tinham excesso de peso, dos quais 650 milhões eram classificados como obesos.^1^ A obesidade é uma doença em que o excesso de gordura corporal se acumula a ponto de comprometer a saúde, sendo caracterizada pela combinação de consumo excessivo de energia, falta de atividade física e predisposição genética.^2^

Nos países ocidentais, a ingestão dietética de gordura (isto é, aproximadamente 40%) excede os valores nutricionais recomendados de 5% a 10%,^3^ e este tipo de dieta pode levar ao desenvolvimento de distúrbios metabólicos, renais, hepáticos, pancreáticos, e cardiovasculares.^4-8^ Essas complicações incluem obesidade, acúmulo de gordura na região abdominal, resistência à insulina, hipertensão, alterações na função cardíaca, desenvolvimento de doença hepática gordurosa não alcoólica, disfunção endotelial e aumento da inflamação e apoptose.^4,7,9-11^

O treinamento resistido (TR) é recomendado como ferramenta não farmacológica para combater e prevenir diversas doenças cardiometabólicas, incluindo a obesidade.^12^ Uma metanálise recente envolvendo estudos clínicos mostrou que o TR pode aumentar a massa corporal magra e reduzir a massa e o percentual de gordura corporal em indivíduos com sobrepeso e obesidade.^13^ Sabe-se que o TR estimula o gasto energético total e promove adaptações no tecido adiposo que potencializam a lipólise e previnem o acúmulo de lipídios.^14^

Roedores (por exemplo, ratos e camundongos) têm sido usados como modelos para estudar os efeitos de dietas hiperlipídicas (DH) (isto é, de 40% a 60% de lipídios) nos parâmetros cardíacos.^15,16^ Sabe-se que a DH leva ao acúmulo de lipídios no coração, que está associado ao aumento do estresse oxidativo, inflamação e apoptose de cardiomiócitos.^17^ Esses efeitos da DH contribuem para alterações funcionais e estruturais no coração e consequente remodelação cardíaca. Nesse sentido, a DH pode aumentar a massa e a fibrose do ventrículo esquerdo (VE), reduzir a fração de ejeção e a fração de encurtamento e aumentar a espessura do VE durante a sístole e a diástole.^9,10,18^

Em relação ao exercício físico, camundongos tratados com DH e submetidos a exercícios aeróbicos (isto é, corrida e natação) demonstraram adaptações positivas no tecido adiposo (isto é, baixo teor lipídico e redução do estresse oxidativo e da inflamação).^19^ No coração, ratos exercitados apresentaram melhorias nos parâmetros estruturais e na capacidade contrátil.^20-23^ Em relação ao TR, ratos com hipertensão arterial sistêmica ou pulmonar submetidos ao TR apresentaram melhora da função cardíaca^24^ e da função contrátil dos miócitos do VE, enquanto o conteúdo de colágeno e a fibrose miocárdica^25^ estavam diminuídos, indicações evidentes de cardioproteção. Apesar disso, os efeitos do TR na estrutura e função cardíaca de roedores alimentados com DH têm sido menos investigados. Portanto, na presente revisão sistemática, nosso objetivo foi avaliar os benefícios do TR no coração de ratos e camundongos alimentados com DH.

## Métodos

### Protocolo e registro

A presente revisão sistemática foi conduzida de acordo com os critérios estabelecidos pelo Preferred Reporting Items for Systematic Review and Meta-Analyses (Itens de Relatório Preferidos para Revisões Sistemáticas e Metanálises – PRISMA). O protocolo desenvolvido foi registrado no International Prospective Register of Systematic Reviews (Registro Prospectivo Internacional de Revisões Sistemáticas – PROSPERO) sob número de registro CRD42022369217.

### Estratégia de pesquisa

Foram identificados estudos relevantes por meio de busca nas bases de dados PubMed, Scopus e Embase dos últimos 15 anos, de dezembro de 2007 a dezembro de 2022. Foram associados os termos descritores em inglês aos operadores booleanos da seguinte forma: (“strength training” OR “resistance training” OR “weight training”) AND (obes* OR “high fat diet” OR HFD) AND (rat OR mice OR mouse) AND (heart OR cardiomyocyte OR cardiac OR “left ventricle”).

### Critérios de eligibilidade

Para a elegibilidade dos estudos, foi aplicada a estratégia PICOS conforme apresentado na Tabela 1. A avaliação dos critérios de elegibilidade foi realizada de forma cega por 2 pesquisadores independentes (AMOP e SFFC). As divergências entre os pesquisadores foram discutidas com um terceiro pesquisador (AJN) e resolvidas em consenso.

**Tabela 1:** – Critérios de população, intervenção, comparação, desfechos e estudo (PICOS)

Critérios de inclusão	Critérios de exclusão
**População**	**População**
Roedores	Estudos em humanos
	Estudos in silico
	Estudos ex vivo
**Intervenção**	**Intervenção**
Treinamento resistido com duração total ≥ 4 semanas	Sem intervenção com treinamento resistido
	Outro tipo de intervenção de exercício
**Comparação**	**Comparação**
• Grupo exercitado em comparação com não exercitado (sedentário), ambos tratados com DH	Grupo sedentário ou exercitado não tratado com DH
**Desfechos**	**Desfechos**
• Estrutura cardíaca, função, estresse oxidativo, inflamação, biogênese mitocondrial e marcadores de remodelamento tecidual	Sem determinação de desfechos em relação ao tecido cardíaco
**Parâmetros de publicação**	**Parâmetros de publicação**
Estudo original	Estudo não original
Publicado entre dezembro de 2007 e dezembro de 2022	Cartas
Língua inglesa	Resumos

DH: dieta hiperlipídica.

### Extração e análise de dados

Os dados e informações de interesse foram extraídos por AMOP, SFFC e LBL. As principais informações obtidas foram sobre características dos animais (espécie, linhagem, sexo e idade); período e composição da DH (% gordura); protocolo de TR (modelo, intensidade, séries, período de descanso, frequência semanal e duração total da intervenção); e resultados antropométricos, metabólicos e cardíacos.

### Avaliação de risco de viés e de qualidade dos estudos

O risco de viés foi examinado de acordo com as diretrizes recomendadas na ferramenta de risco de viés para estudos em animais SYRCLE (Systematic Review Centre for Laboratory Animal Experimentation).^26^ As perguntas foram respondidas com “sim” (baixo risco de viés), “não” (alto risco de viés) ou “incerto” (risco incerto de viés), de acordo com cada um dos 10 itens a seguir: geração de sequência aleatória, características de base, ocultação de alocação, alojamento aleatório, cegamento dos cuidadores/investigadores, avaliação aleatória do desfecho, cegamento da avaliação dos desfechos, dados incompletos dos desfechos, relato seletivo e outros vieses. A avaliação foi realizada de forma cega por 2 pesquisadores independentes (AMOP e LBL). Foi utilizado o software Review Manager, versão 5.4 para realizar essa análise e produzir figuras de risco de viés.

A qualidade dos estudos foi avaliada usando a lista de verificação Collaborative Approach to Meta-Analysis and Review of Animal Data from Experimental Studies (CAMARADES) contendo 10 itens.^27^ Os artigos foram pontuados com 1 ponto para relatar as informações necessárias e zero pontos quando faltavam informações, com pontuação total máxima de 10 pontos, sendo de 1 a 3 pontos considerados de baixa qualidade, 4 a 7 pontos considerados de média qualidade e 8 a 10 pontos considerados de alta qualidade. A avaliação foi realizada de forma independente por 2 autores (AMOP e LBL).

## Resultados

### Seleção de estudos

**Figura 1:**
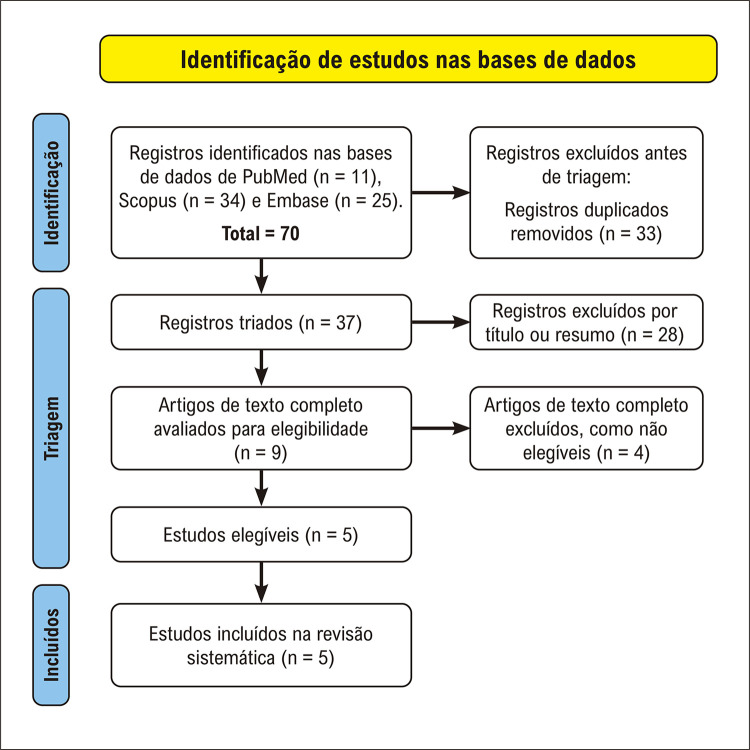
– Fluxograma de busca na literatura.

A busca resultou em 70 artigos (PubMed n = 11; Scopus n = 34; Embase n = 25) (Figura 1). Após exclusão dos artigos duplicados (n = 33), foram selecionados 37 artigos para leitura do título e resumo. Após isso, foram excluídos 28 artigos por não atenderem aos critérios de inclusão: artigos não originais (n = 14); estudos sem modelos animais (n = 1); estudos sem intervenção de TR (n = 11); estudos sem avaliação do coração (n = 1); estudos sem tratamento com DH (n = 1).

Subsequentemente, foram selecionados 9 artigos para leitura do texto completo e avaliação de elegibilidade. Em seguida, foram excluídos 4 artigos: grupo sedentário não tratado com DH (n = 1); sem tratamento com DH (n = 2); e não intervenção com TR (n = 1). Após exclusão, restaram 5 artigos que foram incluídos na revisão sistemática.

### Risco de viés e qualidade dos estudos

**Figura 2:**
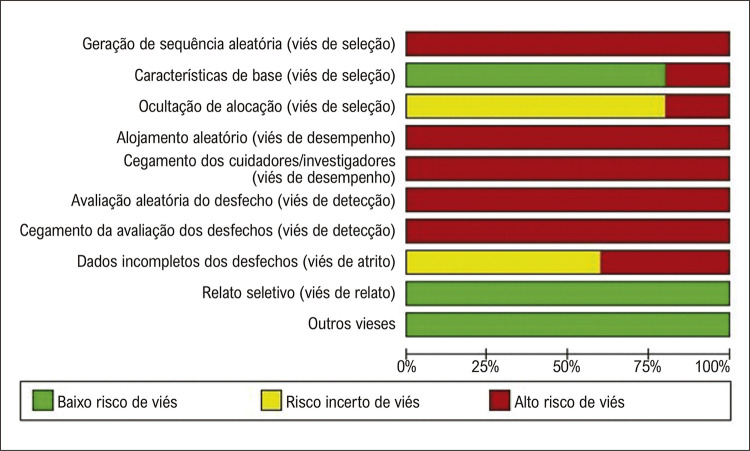
– Quadro de risco de viés, qualidade metodológica e relato dos resultados dos estudos incluídos na revisão sistemática.

Os resultados da análise de viés são apresentados na Figura 2. Em relação ao viés de seleção, o uso de geração de sequência aleatória para reduzir viés de seleção não foi relatado em nenhum dos artigos revisados; portanto, todos os artigos foram classificados como de alto risco de viés. Massa corporal (MC), idade e sexo foram definidos como características basais, e a maioria dos artigos (n = 4; 80%) foi classificada como de baixo risco. Houve um risco incerto de viés para a ocultação de alocação em 4 estudos, que foram classificados como incertos neste item.

Em relação ao viés de desempenho, como nenhum dos artigos relatou se foi utilizado alojamento aleatório ou se os participantes foram cegados (cegamento dos cuidadores/investigadores), todos os artigos receberam alto risco. Para viés de detecção, nenhum dos estudos relatou se houve uma seleção aleatória de animais (avaliação aleatória do desfecho) ou se os avaliadores estavam cegos (cegamento da avaliação dos desfechos); portanto, classificamos todos os artigos como de alto risco. Além disso, 60% dos artigos receberam risco incerto e 40% receberam alto risco devido a dados de resultados incompletos (viés de atrito). Em relação ao relato seletivo (viés de relato) e outros vieses, todos os artigos apresentaram baixo risco de viés.

De acordo com a avaliação CAMARADES (Tabela 2), todos os estudos apresentaram qualidade metodológica média, com pontuações variando de 6 a 7 pontos. Com base nisso, 100% dos estudos foram publicados em periódicos revisados por pares, relataram controle de temperatura, incluíram declaração de conformidade com requisitos regulatórios e utilizaram modelos animais apropriados.

**Tabela 2:** – Avaliação da qualidade metodológica dos estudos incluídos

Estudo	(1)	(2)	(3)	(4)	(5)	(6)	(7)	(8)	(9)	(10)	Pontuação
Lino et al. 2020^28^	☑	☑	☑			☑	☑		☑		6
Effting et al. 2019^31^	☑	☑	☑			☑	☑		☑	☑	7
Kim et al.^33^	☑	☑	☑			☑	☑		☑		6
Leite et al.^29^	☑	☑	☑			☑	☑		☑	☑	7
Melo et al.^30^	☑	☑				☑	☑		☑	☑	6

1) Publicação em periódico revisado por pares; (2) declaração de controle de temperatura; (3) randomização para tratamento ou controle; (4) ocultação de alocação; (5) avaliação cega do desfecho; (6) evitação de anestésicos com propriedades intrínsecas marcantes; (7) modelo animal apropriado; (8) cálculo do tamanho da amostra; (9) declaração de conformidade com requisitos regulatórios; (10) declaração de potenciais conflitos de interesse.

### Características dos animais

Em relação às características dos animais (Tabela 3), a maioria dos estudos (n = 4; 80%) utilizou ratos Wistar^28-30^ e Sprague-Dawley,^30^ enquanto apenas um artigo utilizou camundongos Swiss.^31^ Todos os estudos foram realizados com animais machos, e a idade inicial dos animais variou entre 21 e 90 dias.^28,29,31,32^ Apenas os animais do estudo de Kim et al.^33^ eram mais velhos que os demais (51 semanas).

**Tabela 3:** – Características dos animais, tratamento com dieta hiperlipídica, protocolo de treinamento resistido e principais efeitos cardíacos

Estudo	Características dos animais	Tratamento com DH	Protocolo de TR	Efeitos principais
Lino et al. 2020^28^	Ratos (Wistar) Machos 90 dias de idade	Alimentação com DH (20%) por 11 semanas (3 semanas antes do TR e 8 semanas durante o TR)	**Modelo:** Subida em escada **TCM:** Primeira subida com 75% da MC, com posterior adição de 30 g até falha **Sessões:** 4 subidas com 50%, 75%, 90% e 100% da capacidade máxima de carga. Caso o animal atingisse 100% da carga, foram acrescentados 30 g adicionais para uma nova capacidade máxima de carga com uma quinta subida extra. **Período de descanso entre as séries:** 120 s **Frequência:** 2 dias intercalados por períodos de descanso de 72 horas (3 a 4 vezes/semana) **Duração total:** 8 semanas	**Parâmetros antropométricos e massa gorda:** ↓ MC, ↓ índice de adiposidade **Parâmetros metabólicos e bioquímicos:** ↓ glicemia, ↓ colesterol total ↓ TGL, ↓ HDL, ↔ LDL, ↓ VLDL, ↓ razão de Castelli I e II, ↓ razão TGL/HDL **Propriedades biométricas:** ↔ PC e peso do VE **Estresse oxidativo:** Atividade: ↔ SOD total, ↑ Mn-SOD, ↑ CAT, ↓ GSH, ↔ GPx,↔ peroxidação lipídica **Marcadores de remodelamento tecidual:** Atividade: ↑ Pró-MMP-2, ↑ MMP-2 intermediária, ↔ MMP-2 ativa
Effting et al. 2019^31^	Camundongo (Swiss) Machos 40 dias de idade	Alimentação com DH (59%) por 26 semanas 18 semanas antes do TR e 8 semanas durante o TR)	**Modelo:** Subida em escada **TCM:** Não realizada **Sessões:** 5 a 10 subidas por sessão (progressão de volume) com 20% a 75% da MC (progressão de carga) **Período de descanso entre as séries:** 120 s **Frequência:** Intervalos de 48 horas entre sessões (3 a 4 vezes/semana) **Duração total:** 8 semanas	**Parâmetros antropométricos:** ↓ MC **Parâmetros metabólicos e bioquímicos:** ↓ Glicose em jejum, ↑ taxa de decaimento da glicose no ITT **Estresse oxidativo:** Atividade: ↔ SOD, ↓ CAT Níveis: ↓ DCFH, ↓ MDA, ↔ GSH **Níveis de marcadores inflamatórios:** ↓ TNF-α
Kim et al.^33^	Ratos (Sprague-Dawley) Machos 51 dias de idade	Alimentação com DH (50%) por 18 semanas (6 semanas antes do TR e 12 semanas durante o TR)	**Modelo:** Subida em escada **TCM:** Não realizada **Sessões:** 1 a 8 subidas com 70% a 100% da MC (progressão de carga durante a sessão). Caso o animal conseguisse subir a escada com essas cargas, pesos adicionais eram colocados no cilindro em incrementos de 30 g para cada subida subsequente. **Período de descanso entre as séries:** 120 s **Frequência:** 3 vezes/semana **Duração total:** 12 semanas	**Parâmetros antropométricos e massa gorda:** ↔ MC, ↔ gordura intraperitoneal **Propriedades biométricas:** ↔ PC, ↔ PC/MC **Biogênese mitocondrial** Expressão de proteínas: ↑ Cito-C, ↑ SUD, ↑ PGC1-α, pAMPK/tAMPK. **Marcadores de estresse do retículo endoplasmático:** Expressão de proteínas: ↓ p-PERK/PERK, ↔ CHOP, ↔ GRP78
Leite et al.^29^	Ratos (Wistar) Machos 21 dias de idade	Alimentação com DH (30%) por 24 semanas (12 semanas antes do TR e 12 semanas durante o TR)	**Modelo:** Subida em escada **TCM:** Primeira subida com 75% da MC, com posterior adição de 30 g até falha **Sessões:** 4 subidas com 50%, 75%, 90% e 100% da capacidade máxima de carga. Caso o animal atingisse 100% da carga, era acrescentada uma carga adicional de 30 g até que uma nova carga fosse determinada. **Período de descanso entre as séries:** 120 s **Frequência:** 3 vezes/semana (intervalos de 48 horas) **Duração total:** 12 semanas	**Parâmetros antropométricos e massa gorda:** ↓ MC, ↓ gordura (%), ↑ massa livre de gordura **Propriedades biométricas e hemodinâmicas:** ↓ PAS, ↓ SDB, ↓ PAM, ↔ PC, ↑ PC/MC, ↔ LV **Marcadores de remodelamento tecidual:** Atividade: ↑ Pró-MMP-2, ↑ MMP-2 intermediária, ↔ MMP-2 ativa
Melo et al.^30^	Ratos (Wistar) Machos 37 dias de idade	Alimentação com DH (49.2%) por 26 semanas (16 semanas antes do TR e 10 semanas durante o TR)	**Modelo:** Subida em escada **TCM:** Primeira subida com 50% da MC, com posterior adição de 30 g até falha **Sessões:** Subidas com 50%, 75%, 90% e 100% da capacidade máxima de carga. Caso o animal atingisse 100% da carga, era acrescentada uma carga adicional de 30 g até falha. **Período de descanso entre as séries:** 60 s **Frequência:** 3 vezes/semana (intervalos de 48 horas) **Duração total:** 10 semanas	**Parâmetros antropométricos e massa gorda:** ↔ MC, ↔ gordura corporal, ↔ índice de adiposidade,↔ TABr, ↓ TABe, ↓ TAB visceral **Parâmetros hemodinâmicos:** ↔ PAS, ↔ PAD **Parâmetros metabólicos e bioquímicos:** ↔ Glicose, ↔ colesterol total, ↔ HDL **Propriedades biométricas:** ↔ Coração, ↔ PC/CT, ↔ LV, ↔ LV/CT **Propriedades histológicas:** ↔ AT, ↔ colágeno **Função contrátil dos cardiomiócitos:** ↔ FEC, ↔ tempo até 50% de contração e relaxamento, ↔ taxa máxima de contração e relaxamento **Proteínas envolvidas no manejo de cálcio:** Expressão de proteínas: ↔ Serca2a, ↔ PLB, ↔ pPLBser16, ↔ SERCA2A/PLB,↔ pPLBser16/PLB

↑: aumento; ↓: diminuição; ↔: sem alteração; AMPK: proteína quinase ativada por AMP; AT: área transversal; CAT: catalase; CHOP: proteína pró-apoptótica homóloga a C/EBP; Cito-C: citocromo C; CT: comprimento tibial; DCFH: diclorodihidrofluoresceína; DH: dieta hiperlipídica; FEC: fração de encurtamento; GPx: glutationa peroxidase; GRP78: proteína 78 regulada por glicose; GSH: glutationa oxidase; HDL: lipoproteína de alta densidade; ITT: teste de tolerância à insulina; LDL: lipoproteína de baixa densidade; MC: massa corporal; MDA: malondialdeído; MMP-2: metaloproteinase-2; Mn-SOD: superóxido dismutase mitocondrial; PAD: pressão arterial diastólica; PAM: pressão arterial média; pAMPK: fosfo-AMPK; PAS: pressão arterial sistólica; PC: peso cardíaco; PERK: proteína quinase do retículo endoplasmático semelhante à proteína quinase R; PGC1-α: coativador 1-alfa do receptor gama ativado por proliferador de peroxissomo; PLB: fosfolambam; pPLBser16: fosfolambam fosforilado na serina 16; SERCA2a: Ca^2+^-ATPase do retículo sarcoplasmático; SUD: succinato desidrogenase; TAB: tecido adiposo branco TABe: tecido adiposo branco epididimal; TABr: tecido adiposo branco retroperitoneal; tAMPK: AMPK total; TCM: teste de carga máxima; TGL: triglicerídeos; TNF-α: fator de necrose tumoral alfa; TR: treinamento resistido; VE: ventrículo esquerdo; VLDL: lipoproteína de densidade muito baixa.

### Tratamento com dieta hiperlipídica

O percentual de gordura nas DHs variou entre 20% e 59% (Tabela 3). A duração total do protocolo de alimentação com DH variou entre 11 e 26 semanas. Em todos os estudos, os animais receberam uma DH por um período anterior ao início do TR, que variou entre 3 e 18 semanas.^28,29,31-33^ Subsequentemente, a DH foi mantida durante o período de TR até o final do experimento, que durou 8,^28,32^ 10,^33^e 12 semanas.^29,33^

### Protocolos de treinamento resistido

A Tabela 3 apresenta as características dos protocolos de TR utilizados nos estudos selecionados. Em relação à modalidade de exercício, a subida em escada vertical foi utilizada em todos os estudos. Em 3 estudos (60%), antes do início do TR, os animais foram submetidos ao teste de carga máxima (TCM) para determinação da carga utilizada nas sessões de TR. Nestes estudos, o TCM consistiu em subir escadas com carga equivalente a 50%^32^ e 75%^28,29^ da MC. Nas subidas subsequentes foram acrescentadas cargas de 30 g até o animal atingir a fadiga muscular. Nos demais estudos (n = 2), a MC foi utilizada como base para determinação da carga de TR; portanto, não foi usado o TCM.

Em 3 estudos, a sessão de TR consistiu em 4 subidas com 50%, 75%, 90% e 100% do TCM; caso um animal atingisse 100% do seu TCM, uma carga adicional de 30 g era adicionada até a subida subsequente (sem volume fixo). Nos estudos de Leite et al.^29^ e Lino et al.^28^ foram acrescentadas cargas de 30 g para cada subida até que uma nova carga fosse determinada como carga do TR. Por outro lado, Melo et al.^32^ reajustaram a carga do TR com um novo TCM; porém, os animais iniciaram a primeira subida com o equivalente de 50% da carga da sessão de TR anterior.

Em 2 estudos os animais não foram submetidos ao TCM; portanto, realizaram a subida da escada com peso equivalente à sua MC. No estudo de Effting et al.,^31^ os animais subiram a escada com carga equivalente a 20% da MC nas semanas 1 e 2, 50% da terceira à sexta semana e 75% na sétima e oitava. O número de séries variou durante o protocolo de TR da seguinte forma: nas semanas 1 e 3, os animais realizaram 5 séries; nas semanas 2, 4 e 7 realizaram 7 séries; e nas semanas 5, 7 e 8 realizaram 10 séries. No estudo de Kim et al.,^33^ durante a primeira semana, os animais escalaram com 30% a 50% da sua MC, e os pesos e o número de repetições foram aumentados gradativamente, mas não especificados. A partir da segunda semana até a conclusão do programa de exercícios, a série 1 foi realizada com pesos de 70% da MC, as séries 2 e 3 com pesos de 80%, as séries 4 e 5 com pesos de 90% e as séries 6 a 8 com pesos de 100%. Se um rato conseguisse subir a escada com essas cargas, pesos adicionais eram colocados no cilindro em incrementos de 30 g para cada subida subsequente.

Em apenas um estudo, o período de descanso entre as séries foi de 60 segundos,^33^ enquanto nos demais foi de 120 segundos.^28,29,31,33^ Em 3 estudos, a frequência do TR foi de 3 vezes por semana.^29,32,33^ No estudo de Effting et al.,^31^ as sessões de TR foram separadas por 48 horas; portanto, a frequência semanal variou de 3 a 4 vezes, totalizando 28 sessões. Em Lino et al.,^28^ os ratos realizaram o protocolo de TR durante 2 dias intercalados por períodos de descanso de 72 horas (frequência semanal de 3 a 4 vezes). A duração total da intervenção de TR variou entre 8 e 12 semanas.

### Efeitos principais

Em relação aos parâmetros antropométricos e à massa gorda, o TR reduziu a MC final em 3 estudos,^28,29,31^ acompanhado por uma redução do percentual de gordura corporal e aumento da massa livre de gordura,^29^ embora não tenham sido observadas alterações no índice de adiposidade.^28^ Além disso, em outro estudo, o TR não alterou a MC e o índice de adiposidade, mas reduziu a gordura epididimal e visceral.^32^ No estudo de Kim et al.,^33^ o TR não reduziu a MC final e a gordura intraperitoneal (soma da gordura epididimal, mesentérica e retroperitoneal).

Além disso, o TR melhorou a resistência à insulina,^33^ reduziu a glicemia^28, 33^ e neutralizou o aumento do colesterol total, triglicerídeos, HDL e VLDL induzido pela alimentação com DH.^28^ Também reduziu os seguintes marcadores cardiometabólicos: razão I de Castelli. (colesterol total/HDL), II (LDL/HDL) e a relação triglicerídeos/HDL.^28^ Em outro estudo, a alimentação com DH e o TR não afetaram a glicemia de jejum, marcadores lipídicos (por exemplo, colesterol total e HDL) e pressão arterial sistólico e diastólico.^32^ Por outro lado, no estudo de Leite et al.,^29^ o TR neutralizou o aumento da pressão arterial sistólica, diastólica e média que ocorreu em resposta à alimentação com DH.

Além disso, o TR reduziu os níveis de marcadores inflamatórios (por exemplo, TNF-α) e o estresse oxidativo cardíaco, denotado por níveis mais baixos de DCFH e MDA, apesar da redução da atividade de catalase e da atividade inalterada de SOD.^31^ Em outro estudo,^28^ o TR aumentou a atividade enzimática antioxidante (por exemplo, Mn-SOD e CAT) e reduziu a de GSH e aumentou a atividade de marcadores de remodelamento tecidual (pró-MMP-2 e MMP-2 intermediária). A isoforma ativa da MMP-2 não se alterou após a intervenção de TR.^28^ Os resultados das atividades das isoformas da MMP-2 após a intervenção de TR foram semelhantes aos mostrados por Leite et al.^29^

Nesse sentido, a intervenção de TR aumentou a expressão de proteínas relacionadas à biogênese mitocondrial, como Cito-C, SUD e PGC-1α, e reduziu a expressão dos marcadores de estresse do retículo endoplasmático p-PERK/PERK, embora não tenha alterado a expressão de CHOP e GRP78.^33^ No estudo de Melo et al.,^32^ o TR não modificou o conteúdo de colágeno e a área transversal do VE. Além de não melhorar a função contrátil dos cardiomiócitos (por exemplo, fração de encurtamento, taxa máxima de contração e relaxamento e tempo até 50% de contração e relaxamento), o TR não modificou a expressão de proteínas relacionadas ao manejo de Ca^2^, como Serca2a, PLB e pPLBser16, e as respectivas razões SERCA2A/PLB e pPLBser16/PLB. A maioria dos estudos relatou propriedades biométricas inalteradas (ou seja, peso do coração, massa do VE e suas proporções em relação à MC e ao comprimento tibial) após a intervenção de TR.^28,31,32^ Apenas Leite et al.,^29^ mostraram massa do coração e do VE inalteradas, mas os aumentos no peso cardíaco foram normalizados para MC.

## Discussão

Na presente revisão sistemática, nosso objetivo foi avaliar os benefícios do TR no coração de ratos e camundongos alimentados com DH. Observamos que o TR positivamente afeta os parâmetros antropométricos, metabólicos, funcionais e estruturais alterados pela DH.

Alguns estudos demonstraram que o tratamento com DH compromete a função e a estrutura cardíaca, além de aumentar a inflamação e o estresse oxidativo.^9,10,17,18^ Embora no estudo realizado por de Lino et al.,^28^ a alimentação com DH (20%) durante 11 semanas não tenha afetado a peroxidação lipídica cardíaca e a atividade enzimática antioxidante (por exemplo, SOD, Mn-SOD, CAT e GPx) em ratos, a intervenção com RT provou exercer uma função cardioprotetora. Nesse sentido, após 8 semanas de TR, os animais apresentaram menor MC e índice de adiposidade, maiores atividades cardíacas de Mn-SOD e CAT e menor GSH.^28^ Esses achados estão de acordo com outros estudos nos quais foi observado aumento na capacidade antioxidante cardíaca em ratos com hipertensão renovascular submetidos ao treinamento de força.^34^

Nesse contexto, Effting et al.,^31^ mostraram que o TR foi eficaz na redução da MC e na melhora do metabolismo da glicose, além de neutralizar o aumento do estresse oxidativo (por exemplo, aumento de DCFH e MDA) e dos marcadores inflamatórios (por exemplo, TNF-α) nos corações de camundongos alimentados com DH (59% por 26 semanas). O aumento do TNF-α após a alimentação com DH pode ser explicado pela capacidade das espécies reativas de oxigênio de promover lesões no tecido cardíaco, o que pode ter levado a alterações nas respostas imunes.^32^ Sabe-se que o estresse oxidativo desencadeia a hipertrofia cardíaca patológica e compromete a função contrátil dos cardiomiócitos.^35,36^ Com base nisso, os mecanismos antioxidantes enzimáticos cardíacos são essenciais para restaurar o estado redox e evitar o acúmulo exacerbado de agentes pró-oxidantes que contribuem para a deterioração cardíaca.^37^ Lino et al.^28^ mostraram que o RT regulou positivamente Mn-SOD e CAT. Entretanto, o estudo de Effting et al.^31^ indicou que a atividade do SOD não se alterou e a da CAT foi negativamente regulada pela TR, sugerindo que outros mecanismos estão relacionados à antioxidação, o que requer investigação mais aprofundada.

No estudo de Kim et al.,^33^ ratos tratados com DH (50%) foram submetidos a 12 semanas de TR e apresentaram redução nos marcadores de estresse do retículo endoplasmático (por exemplo, pPERK/PERK) no VE. Porém, no mesmo estudo, a expressão do CHOP, outro marcador de estresse do retículo endoplasmático, foi reduzida apenas no grupo que realizou exercício aeróbio, o que foi associado à redução da MC observada apenas neste modelo de treinamento, denotando a importância do controle antropométrico.^33^ Considerando que o estresse do retículo endoplasmático está associado ao estresse oxidativo e à inflamação e está aumentado na hipertrofia cardíaca patológica e na insuficiência cardíaca,^38^ esses achados indicam cardioproteção induzida pelo TR. Além disso, o TR melhorou a biogênese mitocondrial do VE, conforme evidenciado pelo aumento da expressão de Cito-C, SUD e PGC-1α. Tais achados indicam os efeitos benéficos do TR, uma vez que o aumento da biogênese mitocondrial está associado à redução do estresse oxidativo e da apoptose.^39^ A biogênese mitocondrial também conferiu proteção miocárdica em um modelo de insuficiência cardíaca.^40^

Em relação ao remodelamento estrutural cardíaco e da matriz extracelular, em dois estudos,^28,29^ o TR aumentou a atividade da MMP-2 no VE de ratos alimentados com DH. Sabe-se que a MMP-2 expressa em cardiomiócitos atua diretamente na renovação da matriz extracelular, promovendo a degradação de componentes como colágeno e fibronectina.^41^ Guzzoni et al.^42^ demonstraram que 12 semanas de TR aumentaram a atividade da MMP-2 ativa, com consequente redução do seu inibidor endógeno TIMP-1, o que contribuiu diretamente para a neutralização do aumento de colágeno e fibrose no VE em resposta ao envelhecimento. Como vários estudos indicam que a DH aumenta os níveis de colágeno e a fibrose cardíaca,^33^ a regulação positiva das isoformas da MMP-2 em resposta ao TR é importante para mitigar os danos às propriedades funcionais do coração.

Na maioria desses estudos, a DH e o TR não afetaram diretamente as propriedades biométricas cardíacas, que são marcadores indiretos de hipertrofia cardíaca. Por exemplo, as massas do coração e do VE,^28,29,31,32^ bem como suas proporções em relação ao comprimento tibial,^33^ permaneceram inalteradas. Somente no estudo de Leite et al.,^29^ o TR aumentou a razão coração/MC no grupo tratado com DH, que diminuiu em resposta ao TR. Outro estudo com ratos saudáveis também mostrou que o TR aumenta a relação entre VE e MC.^30^

É bem compreendido que, a longo prazo, o TR promove hipertrofia cardíaca concêntrica fisiológica, caracterizada pela adição de sarcômeros em paralelo, aumento da massa cardíaca e aumento da espessura da parede do VE.^43^ Tais adaptações melhoraram a função contrátil cardíaca ao nível celular e dos órgãos, sem a presença de alterações deletérias (ou seja, aumento de tecido fibrótico, estresse oxidativo, apoptose e inflamação).^43^ Por outro lado, nos estudos incluídos, o TR não alterou o coração e a massa do VE,^28,29,31,33^ ou a área transversal e o conteúdo de colágeno do VE^33^ em animais alimentados com DH e dieta de ração comercial. Com base nisso, são necessários mais estudos que avaliem as alterações histomorfométricas e as vias de sinalização envolvidas no remodelamento estrutural cardíaco para explorar os efeitos do TR nesse modelo.

Em relação à função dos cardiomiócitos, Melo et al.^32^ demonstraram que o TR melhorou a função contrátil dos cardiomiócitos em ratos alimentados com dieta de ração comercial. Embora a DH (49,2% de gordura durante 26 semanas) não tenha afetado negativamente a função contrátil dos cardiomiócitos (por exemplo, fração de encurtamento, velocidades de contração e relaxamento), o efeito benéfico do TR também não foi observado nesses animais. Esses resultados sugerem que ratos alimentados com DH apresentam resistência aos efeitos benéficos do TR. Outros estudos demonstraram que o TR melhorou a função contrátil dos cardiomiócitos em ratos com doença cardiovascular.^25^ Além disso, Lavorato et al.^44^ relataram que 8 semanas de corrida em esteira aumentaram a função contrátil e o cálcio intracelular transitório em cardiomiócitos, enquanto a DH (53%) comprometeu esses parâmetros. Mais estudos são necessários para explorar o efeito do TR na função dos cardiomiócitos em animais tratados com DH.

A presente revisão tem algumas limitações. Primeiro, poucos estudos examinaram os efeitos do TR na DH; portanto, mais estudos nesta área devem ser realizados para compreender os mecanismos celulares e moleculares do coração. Em segundo lugar, a duração da alimentação e o percentual de gordura na DH foram diferentes entre os estudos, o que dificulta a observação homogênea dos efeitos do tratamento com DH. Por exemplo, o estudo de Melo et al.^32^ incluído na revisão sistemática demonstrou que 26 semanas de tratamento com DH (49,2%) não alteraram a área transversal dos cardiomiócitos. Porém, foi demonstrado um resultado oposto em outro estudo que utilizou uma dieta com maior percentual de gordura (60%) durante 20 semanas.^45^

Nesse sentido, é importante destacar o fato de terem sido incluídos estudos com diferentes espécies (isto é, rato e camundongo) e linhagens (isto é, Wistar, Sprague-Dawley e Swiss) de roedores, o que pode influenciar os resultados gerais dos efeitos da DH. Gong et al.^46^ revelaram que camundongos alimentados com DH (45% kcal de gordura por 16 semanas) apresentaram hipertrofia cardíaca ao nível do órgão (massa do coração e do VE) e ao nível celular (aumento da área transversal), o que não foi observado nos estudos incluídos. Além disso, o mesmo estudo de Gong et al.^46^ relatou comprometimento da função contrátil cardíaca (fração de encurtamento e tempo até 90% de relaxamento prolongado), diferentemente dos achados de Melo et al.^32^ Além disso, apenas um estudo avaliou resultados relacionados à função dos cardiomiócitos, à histomorfometria cardíaca, à biogênese mitocondrial e ao estresse do retículo endoplasmático, que requerem investigações adicionais.

Apesar disso, nosso estudo revela que os modelos de TR e DH não foram extensivamente explorados, e tais lacunas contribuem para o direcionamento de estudos futuros sobre a fisiopatologia cardíaca. Nesse contexto, é importante mencionar que o percentual de gordura presente na DH e o tempo de exposição são fatores fundamentais a serem considerados para esclarecer os reais efeitos da dieta no tecido cardíaco, o que possibilita comparar diferentes intervenções usando um modelo fortemente estabelecido.

Além disso, sugerimos que estudos futuros envolvendo a prática de TR sejam explorados com diferentes cargas de treinamento (ou seja, intensidade, volume, densidade e frequência semanal) para determinar qual modelo parece ser mais adequado para os pacientes. Além disso, é de extrema importância que estudos subsequentes avaliem os desfechos de interesse apresentados nos estudos incluídos nesta revisão sistemática.

## Conclusão

Nossos resultados indicam que o TR parcialmente neutraliza o remodelamento cardíaco adverso induzido pela DH, aumentando a atividade dos marcadores de remodelamento estrutural; elevando a biogênese mitocondrial; reduzindo o estresse oxidativo, marcadores inflamatórios e estresse do retículo endoplasmático; e melhorando os parâmetros hemodinâmicos, antropométricos e metabólicos.
