# Supplementary material for: O Treinamento Resistido Atenua a Remodelação Cardíaca Induzida por uma Dieta Hiperlipídica em Roedores: Uma Revisão Sistemática
Source: Arq Bras Cardiol. 2024 Apr 11;121(4):e20230490. [Article in Portuguese] doi: 10.36660/abc.20230490 (PMC11098569; doi:10.36660/abc.20230490)
Supplement: Translation

# Translation: Resistance Exercise Training Mitigates Cardiac Remodeling Induced by a High-Fat Diet in Rodents: A Systematic Review

**DOI:** 10.36660/abc.20230490i

**Authors:** Alexandre Martins Oliveira Portes, Sebastião Felipe Ferreira Costa, Luciano Bernardes Leite, Victor Neiva Lavorato, Denise Coutinho de Miranda, Anselmo Gomes de Moura, Leôncio Lopes Soares, Mauro César Isoldi, Antônio José Natali

**Affiliations:** 1 Brazil Universidade Federal de Viçosa, Viçosa, MG – Brazil; 2 Brazil Universidade Federal de Ouro Preto – Campus Morro do Cruzeiro, Ouro Preto, MG – Brazil

**Keywords:** Cardiac Myocytes, High-Fat Diet, esistance Training, Systematic Review

## Abstract

**Background:**

Obesity is associated with the development of cardiovascular diseases and is a serious public health problem. In animal models, high-fat diet (HFD) feeding impairs cardiac structure and function and promotes oxidative stress and apoptosis. Resistance exercise training (RT), however, has been recommended as coadjutant in the treatment of cardiometabolic diseases, including obesity, because it increases energy expenditure and stimulates lipolysis.

**Objective:**

In this systematic review, we aimed to assess the benefits of RT on the heart of rats and mice fed HFD.

**Methods:**

Original studies were identified by searching PubMed, Scopus, and Embase databases from December 2007 to December 2022. This study was conducted in accordance with the criteria established by PRISMA and registered in PROSPERO (CRD42022369217). The risk of bias and methodological quality was evaluated by SYRCLE and CAMARADES, respectively. Eligible studies included original articles published in English that evaluated cardiac outcomes in rodents submitted to over 4 weeks of RT and controlled by a sedentary, HFD-fed control group (n = 5).

**Results:**

The results showed that RT mitigates cardiac oxidative stress, inflammation, and endoplasmic reticulum stress. It also modifies the activity of structural remodeling markers, although it does not alter biometric parameters, histomorphometric parameters, or the contractile function of cardiomyocytes.

**Conclusion:**

Our results indicate that RT partially counteracts the HFD-induced adverse cardiac remodeling by increasing the activity of structural remodeling markers; elevating mitochondrial biogenesis; reducing oxidative stress, inflammatory markers, and endoplasmic reticulum stress; and improving hemodynamic, anthropometric, and metabolic parameters.

## Introduction

Currently, obesity is a serious public health problem. In 2016, more than 1.9 billion adults were overweight, 650 million of whom were classified as obese.^1^ Obesity is a disease in which excess body fat accumulates to the point of affecting health. This is characterized by the combination of excessive energy consumption, lack of physical activity, and genetic predisposition.^2^

In Western countries, the dietary intake of fats (i.e., approximately 40%) exceeds the recommended nutritional values of 5% to 10%,^3^ and this type of diet can lead to the development of metabolic, renal, hepatic, pancreatic, and cardiovascular disorders.^4-8^ These complications include obesity, accumulation of fat in the abdominal region, insulin resistance, hypertension, changes in cardiac function, development of non-alcoholic fatty liver disease, endothelial dysfunction, and increased inflammation and apoptosis.^4,7,9-11^

Resistance exercise training (RT) is recommended as a non-pharmacological tool to combat and prevent several cardiometabolic diseases, including obesity.^12^ A recent meta-analysis involving clinical studies showed that RT can increase lean body mass and reduce body fat mass and percentage in overweight and obese individuals.^13^ It is known that RT stimulates total energy expenditure and promotes adaptations in the adipose tissue that enhance lipolysis and prevent the accumulation of lipids.^14^

Rodents (e.g., rats and mice) have been used as models to study the effects of high-fat diets (HFD) (i.e., 40% to 60% lipids) on cardiac parameters.^15,16^ It is known that HFD leads to the accumulation of lipids in the heart, which is associated with increased oxidative stress, inflammation, and apoptosis of cardiomyocytes.^17^ These effects of HFD contribute to functional and structural changes in the heart and consequent cardiac remodeling. In this sense, HFD can increase left ventricular (LV) mass and fibrosis, reduce ejection fraction and fractional shortening, and increase the thickness of the LV during systole and diastole.^9,10,18^

Regarding physical exercise, mice treated with HFD and submitted to aerobic exercise (i.e., running and swimming) have shown positive adaptations in the adipose tissue (i.e., low lipid content and reduced oxidative stress and inflammation).^19^ In the heart, exercised rats presented improvements in structural parameters and contractile capacity.^20-23^ Concerning RT, rats with systemic or pulmonary arterial hypertension submitted to RT showed improved cardiac function^24^ and LV myocyte contractile function, while collagen content and myocardial fibrosis^25^ were diminished, which are clear indications of cardioprotection. Despite that, the effects of RT on the cardiac structure and function of rodents fed HFD have been less investigated. Therefore, in this systematic review we aimed to assess the benefits of RT on the hearts of rats and mice fed HFD.

## Methods

### Protocol and registration

This systematic review was conducted in accordance with the criteria established by the Preferred Reporting Items for Systematic Review and Meta-Analyses (PRISMA). The developed protocol was registered in the International Prospective Register of Systematic Reviews (PROSPERO) under registration number CRD42022369217.

### Search strategy

Relevant studies were identified by searching PubMed, Scopus, and Embase databases from the last 15 years from December 2007 to December 2022. The descriptor terms were associated with the Boolean operators as follows: (“strength training” OR “resistance training” OR “weight training”) AND (obes* OR “high fat diet” OR HFD) AND (rat OR mice OR mouse) AND (heart OR cardiomyocyte OR cardiac OR “left ventricle”).

### Eligibility criteria

For the studies eligibility, the PICOS strategy was applied as displayed in Table 1. The evaluation of the eligibility criteria was performed blindly by 2 independent researchers (AMOP and SFFC). Disagreements between researchers were discussed with a third researcher (AJN) and resolved in consensus.

**Table 1:** – Population, intervention, comparison, outcomes, and study (PICOS) criteria

Inclusion criteria	Exclusion criteria
**Population**	**Population**
Rodents	Humans
	In silico studies
	Ex vivo studies
**Intervention**	**Intervention**
Resistance training with total duration ≥ 4 weeks	No intervention with resistance training
	Other type of exercise intervention
**Comparators**	**Comparators**
Exercised group compared with non-exercised (sedentary), both treated with HFD	Sedentary or exercised group not treated with HFD
**Outcomes**	**Outcomes**
Cardiac structure, function, oxidative stress, inflammation, mitochondrial biogenesis, and markers of tissue remodeling	No determination of outcomes with respect to cardiac tissue
**Publication parameters**	**Publication parameters**
Original study	Non-original study
Published between December 2007 and December 2022	Letters
English language	Abstracts

HFD: high-fat diet.

### Data extraction and analysis

Data and information of interest were extracted by AMOP, SFFC, and LBL. The main information obtained were on animal characteristics (lineage, strain, sex, and age); period and composition of HFD treatment (% fat); RT protocol (model, intensity, series, rest period, weekly frequency, and total duration of intervention); and anthropometric, metabolic, and cardiac outcomes.

### Risk of bias and study quality evaluation

The risk of bias was examined conforming to the guidelines recommended in the risk of bias tool for animal studies SYRCLE (Systematic Review Centre for Laboratory Animal Experimentation).^26^ The questions were answered with “yes” (low risk of bias), “no” (high risk of bias), or “unclear” (unclear risk of bias), according to each of the following 10 item tools: random sequence generation, baseline characteristics, allocation concealment, random housing, blinding of caregivers/investigators, random outcome assessment, blinding of outcome assessment, incomplete outcome data, selective reporting, and other bias. The evaluation was performed blindly by 2 independent researchers (AMOP and LBL). Review Manager software, version 5.4 was used to conduct this analysis and to produce the risk of bias figures.

The quality of studies was evaluated using the Collaborative Approach to Meta-Analysis and Review of Animal Data from Experimental Studies (CAMARADES) checklist containing 10 items.^27^ The articles were scored with one point for reporting the required information and zero points when information was missing, with a total score of maximum 10 points, with 1 to 3 points regarded as low quality, 4 to 7 points regarded as medium quality, and 8 to 10 points regarded as high quality. The evaluation was performed independently by 2 authors (AMOP and LBL).

## Results

### Selection of studies

The search resulted in 70 articles (PubMed n = 11; Scopus n = 34; Embase n = 25) (Figure 1). After excluding duplicate articles (n = 33), 37 articles were selected for the title and abstract reading. After that, 28 articles were excluded for not meeting the inclusion criteria: non-original articles (n = 14); studies without animal models (n = 1); studies without RT intervention (n = 11); studies without evaluation of the heart (n = 1); no treatment with HFD (n = 1).

**Figure 1:** – Flow diagram for literature search.

Subsequently, 9 articles were selected for full text reading and eligibility assessment. Then, 4 articles were excluded: sedentary group not treated with HFD (n = 1); non-treatment with HFD (n = 2); and non-intervention with RT (n = 1). After exclusion, 5 articles remained and were included in the systematic review.

### Risk of bias and study quality

The results from bias analysis are shown in Figure 2. For selection bias, the use of random sequence generation for reducing selection bias was not reported in any of the reviewed articles; therefore, all articles were graded as high risk of bias. Body mass (BM), age, and sex were defined as the baseline characteristics, and the majority of the articles (n = 4; 80%) were graded as low risk. There was an unclear risk of bias for allocation concealment in 4 studies, which were thus graded as unclear on this item.

**Figure 2:** – Risk of bias chart of methodological quality and reporting of results of studies included in the systematic review.

Regarding performance bias, because none of the articles reported whether random housing was used or whether the participants were blinded (blinding of caregivers/investigators), all articles received high risk. For detection bias, none of the studies reported whether there was a random selection of animals (random outcome assessment) or whether evaluators were blinded (blinding of outcome assessment); thus, we graded all articles as high risk. Additionally, 60% of articles received unclear risk, and 40% received high risk for incomplete outcome data (attrition bias). In relation to selective reporting (reporting bias) and other bias, all articles had low risk of bias.

According to the CAMARADES evaluation (Table 2), all studies presented medium methodological quality, with scores ranging from 6 to 7 points. Based on this, 100% of the studies were published in peer-reviewed journals, reported control of temperature, included statement of compliance with regulatory requirements, and used appropriate animal models.

**Table 2:** – Assessment of the methodological quality of the included studies

Study	(1)	(2)	(3)	(4)	(5)	(6)	(7)	(8)	(9)	(10)	Score
Lino et al. 2020^28^	☑	☑	☑			☑	☑		☑		6
Effting et al. 2019^31^	☑	☑	☑			☑	☑		☑	☑	7
Kim et al.^33^	☑	☑	☑			☑	☑		☑		6
Leite et al.^29^	☑	☑	☑			☑	☑		☑	☑	7
Melo et al.^30^	☑	☑				☑	☑		☑	☑	6

(1) Publication in peer-reviewed journal; (2) statement of control of temperature; (3) randomization of treatment or control; (4) allocation concealment; (5) blinded assessment of outcome; (6) avoidance of anesthetics with marked intrinsic properties; (7) appropriate animal model; (8) sample size calculation; (9) statement of compliance with regulatory requirements; (10) statement regarding possible conflict of interest.

Most studies (80%) did not describe the use of any anesthetic; only one study used anesthetic, but not to assess outcomes of interest; therefore, all studies scored 1 point. Two studies did not include a statement of potential conflict of interests, and only one did not present randomly allocated groups. None of the studies reported allocation concealment or blind evaluation of outcome, and none described sample size calculation.

### Animal characteristics

Regarding the characteristics of the animals (Table 3), most studies (n = 4; 80%) used Wistar^28-30^ and Sprague-Dawley rats,^30^while only one article used Swiss mice.^32^All studies were performed with male sex, and the initial age of the animals varied between 21 and 90 days.^28,29,31,32^ Only those in the study by Kim et al.^33^were older than the others (51 weeks).

**Table 3:** – Animal characteristics, high-fat diet treatment, resistance training protocol, and main effects on heart

Study	Animal characteristics	HFD treatment	Resistance training protocol	Main effects
Lino et al. 2020^28^	Rats (Wistar) Male 90 days of age	Feeding with HFD (20%) for 11 weeks (3 weeks before RT and 8 weeks during RT)	**Model:** Ladder climbing **MLCT:** First climbing with 75% of BM and subsequent addition of 30 g until failure **Sessions:** 4 climbings with 50%, 75%, 90%, and 100% of the maximum carrying capacity. If the rat reached 100% of the carrying load, an additional 30 g were added for a new maximum carrying capacity with a fifth extra climbing. **Rest period between sets:** 120 s **Frequency:** Two days interlarded by rest periods of 72 h (3 to 4 times/week) **Total duration:** 8 weeks	**Anthropometric parameters and fat mass:** ↓ BM, ↓ adiposity index **Metabolic and biochemical parameters:** ↓ Glycaemia, ↓ total cholesterol ↓ TGL, ↓ HDL, ↔ LDL, ↓ VLDL, ↓ rCastelli’s ratio I and II, ↓ TGL/HDL ratio **Biometric properties:** ↔ HW and LV weight **Oxidative stress:** Activity: ↔ Total-SOD, ↑ Mn-SOD, ↑ CAT, ↓ GSH, ↔ GPx, ↔ lipid peroxidation **Tissue remodeling markers:** Activity: ↑ Pro MMP-2, ↑ intermediate MMP-2, ↔ active MMP-2
Effting et al. 2019^31^	• Mice (Swiss) • Male • 40 days of age	• Feeding with HFD (59%) for 26 weeks (18 weeks before RT and 8 weeks during RT)	**Model:** Ladder climbing **MLCT:** Not performed **Sessions:** 5 to 10 climbings per session (volume progression) with 20% to 75% of BM (load progression) **Rest period between sets:** 120 s **Frequency:** 48-hour intervals between sessions (3 to 4 times/week) **Total duration:** 8 weeks	**Anthropometric parameters:** ↓ BM **Metabolic and biochemical parameters:** ↓ Fasting glucose, ↑ glucose decay rate in ITT **Oxidative stress:** Activity: ↔ SOD, ↓ CAT Levels: ↓ DCFH, ↓ MDA, ↔ GSH **Inflammatory marker levels:** ↓ TNF-α
Kim et al.^33^	Rats (Sprague-Dawley) Male 51 days of age	Feeding with HFD (50%) for 18 weeks (6 weeks before RT and 12 weeks during RT)	**Model:** Ladder climbing **MLCT:** Not performed **Sessions:** 1 to 8 climbings with 70% to 100% of BM (load progression during the session). If a rat was able to climb the ladder with these loads, additional weights were placed in the cylinder in 30 g increments for each subsequent climbing. **Rest period between sets:** 120 s **Frequency:** 3 times/week **Total duration:** 12 weeks	**Anthropometric parameters and fat mass:** ↔ BM, ↔ intraperitoneal fat **Biometric properties:** ↔ HW, ↔ HW/BM **Mitochondrial biogenesis:** Protein expression: ↑ Cyto-C, ↑ SUD, ↑ PGC1-α, pAMPK/tAMPK. **Endoplasmic reticulum stress markers:** Protein expression: ↓ p-PERK/PERK, ↔ CHOP, ↔ GRP78
Leite et al.^29^	Rats (Wistar) Male 21 days of age	Feeding with HFD (30%) for 24 weeks (12 weeks before RT and 12 weeks during RT)	**Model:** Ladder climbing **MLCT:** First climbing with 75% of BM, with subsequent additional 30 g loads until failure **Sessions:** Four climbings with 50%, 75%, 90%, and 100% of the maximum carrying capacity. If a rat reached 100% of its carrying load, an additional 30-g load was added until a new carrying load was determined. **Rest period between sets:** 120 s **Frequency:** 3 times/week (48-hour intervals) **Total duration:** 12 weeks	**Anthropometric parameters and fat mass:** ↓ BM, ↓ fat (%), ↑ fat-free mass **Biometric and hemodynamic properties:** ↓ SBP, ↓ SDB, ↓ MBP, ↔ HW, ↑ HW/BM, ↔ LV **Tissue remodeling markers:** Activity: ↑ Pro-MMP-2, ↑ intermediate MMP-2, ↔ active MMP-2
Melo et al.^30^	Rats (Wistar) Male 37 days of age	Feeding with HFD (49.2%) for 26 weeks (16 weeks before RT and 10 weeks during RT)	**Model:** Ladder climbing **MLCT:** First climbing with 50% of BM, with subsequent additional 30-g loads until to failure **Sessions:** Climbings with 50%, 75%, 90%, and 100% of the maximum carrying capacity. If the animal reached 100% of the carrying load, an additional 30-g load was added until failure. **Rest period between sets:** 60 s **Frequency:** 3 times/week (48-hour intervals) **Total duration:** 10 weeks	**Anthropometric parameters and fat mass:** ↔ BM, ↔ body fat, ↔ adiposity index, ↔ rWAT, ↓ eWAT, ↓ visceral WAT **Hemodynamic parameters:** ↔ SBP, ↔ DBP **Metabolic and biochemical parameters:** ↔ Glucose, ↔ total cholesterol, ↔ HDL **Biometric properties:** ↔ Heart, ↔ HW/TL, ↔ LV, ↔ LV/TL **Histological properties:** ↔ CSA, ↔ collagen **Cardiomyocyte contractile function:** ↔ FS, ↔ time to 50% of contraction and relaxation, ↔ maximum rate of contraction and relaxation **Calcium handling proteins:** Protein expression: ↔ Serca2a, ↔ PLB, ↔ pPLBser16, ↔ SERCA2A/PLB, ↔ pPLBser16/PLB

↑: ncrease; ↓: decrease; ↔: unchanged; AMPK: AMP-activated protein kinase; BM: body mass; CAT: catalase; CHOP: proapoptotic C/EBP homologous protein; CSA: cross-sectional area; Cyto-C: cytochrome C; DBP: diastolic blood pressure; DCFH: dichlorodihydrofluorescein; eWAT: epididymal white adipose tissue; FS: fractional shortening; GPx: glutathione peroxidase; GRP78: glucose-regulated protein 78; GSH: glutathione oxidase; HDL: high-density lipoprotein; HFD: high-fat diet; HW: heart weight; ITT: insulin tolerance test; LDL: low-density lipoprotein; LV: left ventricle; MBP: mean blood pressure; MLCT: maximal load carrying test; MDA: malondialdehyde; MMP-2: metalloproteinase-2; Mn-SOD: mitochondrial superoxide dismutase; pAMPK: phospho-AMPK; PERK: PKR-like endoplasmic reticulum kinase; PGC1-α: peroxisome proliferator-activated receptor-γ coactivator 1-α; PLB: phospholamban; pPLBser16: PLB serine16 phosphorylation; RT: resistance training; rWAT: retroperitoneal white adipose tissue; SBP: systolic blood pressure; SERCA2a: sarcoplasmic reticulum Ca^2+^ ATPase; SUD: succinate dehydrogenase; tAMPK: total AMPK; TGL: triglycerides; TL: tibial length; TNF-α: tumor necrosis factor-alpha; VLDL: very-low density lipoprotein; WAT: white adipose tissue.

### High-fat diet treatment

The fat percentage of HFD ranged between 20% and 59% (Table 3). The total duration of the HFD feeding protocol ranged between 11 and 26 weeks. In all studies, the animals received HFD for a period before the start of RT, which varied between 3 and 18 weeks.^28,29,31-33^ The HFD was subsequently maintained during the RT period until the end of the experiment, which lasted 8,^28,32^ 10,^33^and 12 weeks.^29,33^

### Resistance training protocols

Table 3 shows the characteristics of the RT protocols used in the selected studies. Regarding exercise mode, climbing a vertical ladder was used in all studies. In 3 studies (60%), prior to the beginning of RT, the animals were submitted to maximal load carrying test (MLCT) to determine the load used in the RT sessions. In these studies, MLCT consisted of climbing the stairs with a load equivalent to 50%^32^and 75%^28,29^of BM, and in subsequent climbs, 30-g loads were added until the point at which the animal reached muscle fatigue. In the other studies (n = 2), BM was used as the basis for determining the RT load; therefore, MLCT was not used.

In 3 studies, the RT session consisted of 4 climbs with 50%, 75%, 90%, and 100% of the MLCT; if a rat reached 100% of its MLCT, an additional 30-g load was added until subsequent climb (without fixed volume). In studies by Leite et al.^29^and de Souza Lino et al.,^28^30-g loads were added for each climb until a new carrying load was determined as the RT load. On the other hand, Melo et al.^32^readjusted the RT load with a new MLCT; however, the animals started the first climb with the equivalent of 50% of the carrying load from the prior RT session.

In 2 studies, the animals were not submitted to the MLCT; therefore, they performed the stair climbing with weight equivalent to their BM. In the study by Effting et al.,^31^ the animals climbed the ladder with a load equivalent to 20% of BM in weeks 1 and 2, 50% from the third to the sixth week, and 75% in the seventh and eighth. The number of series varied during the RT protocol as follows: in weeks 1 and 3, the animals performed 5 series; in weeks 2, 4, and 7, they performed 7 series; and in weeks 5, 7, and 8, they performed 10 series. In the study by Kim et al.,^33^ in the first week the animals climbed with 30% to 50% of their BM, and the weights and the number of repetitions were incrementally increased, but not specified. From the second week until the completion of the exercise program, set 1 was conducted with weights of 70% of BM, sets 2 and 3 with weights of 80%, sets 4 and 5 with weights of 90%, and sets 6 to 8 with weights of 100%. If a rat was able to climb the ladder with these loads, additional weights were placed in the cylinder in 30-g increments for each subsequent climb.

In only one study, the rest period between sets was 60 seconds,^33^ while in the others 120 seconds were used.^28,29,31,33^ In 3 studies, the frequency of RT was 3 times a week.^29,32,33^In the study Effting et al.,^31^ RT sessions were separated by 48 hours; therefore, the weekly frequency ranged from 3 to 4 times, totaling 28 sessions. In de Souza Lino et al.,^28^the rats performed the RT protocol for 2 days interlarded by rest periods of 72 hours (weekly frequency from 3 to 4). The total duration of the RT intervention ranged between 8 and 12 weeks.

### Main effects

Regarding anthropometric parameters and fat mass, RT reduced the final BM in 3 studies,^28,29,31^ which followed a reduction in body fat percentage and increase in fat-free mass,^29^ although no changes in the adiposity index were observed.^28^ Furthermore, in another study, RT did not change the BM and adiposity index, but reduced epididymal and visceral fat.^32^ In the study by Kim et al.,^33^RT did not reduce the final BM and intraperitoneal fat (sum of epididymal, mesenteric, and retroperitoneal fats).

Additionally, RT improved insulin resistance,^33^ reduced blood glucose,^28, 33^ and counteracted the increase in total cholesterol, triglycerides, HDL, and VLDL induced by HFD feeding.^28^ Additionally, it reduced the cardiometabolic markers Castelli’s ratio I (total cholesterol/HDL), II (LDL/HDL), and the triglycerides/HDL ratio.^28^ In another study, HFD feeding and RT did not affect fasting glucose, lipid markers (e.g., total cholesterol and HDL), and systolic and diastolic blood pressure.^32^On the other hand, in the study by Leite et al.,^29^ RT neutralized the increase in systolic, diastolic, and mean blood pressure in response to HFD feeding.

Furthermore, RT reduced levels of inflammatory markers (e.g., TNF-α) and cardiac oxidative stress, denoted by lower levels of DCFH and MDA, despite reduced catalase activity and unchanged SOD activity.^31^In another study,^28^ RT increased antioxidant enzymatic activity (e.g., Mn-SOD and CAT) and reduced that of GSH and increased the activity of tissue remodeling markers (pro- and intermediate MMP-2). The active MMP-2 isoform did not change after the RT intervention.^28^ The results of the activities of the MMP-2 isoforms after RT intervention were similar to those shown by Leite et al.^29^

In this sense, RT intervention increased the expression of proteins related to mitochondrial biogenesis, such as Cyto-C, SUD, and PGC-1α, and reduced the expression of endoplasmic reticulum (ER) stress markers p-PERK/PERK, although it did not change the expression of CHOP and GRP78.^33^In the study by Melo et al.,^32^RT did not modify the collagen content and cross-sectional area of the LV. In addition to not improving the contractile function of cardiomyocytes (e.g., fractional shortening, maximum rate of contraction and relaxation, and time to 50% of contraction and relaxation), RT did not modify the expression of proteins related to Ca^2^ handling, such as Serca2a, PLB, and pPLBser16, and the respective SERCA2A/PLB and pPLBser16/PLB ratios. Most of the studies reported unchanged biometric properties (i.e., heart weight, LV mass, and their ratios to BM and tibial length) following RT intervention.^28,31,32^Only Leite et al.,^29^ showed unchanged heart and LV mass, but increases in heart rate were normalized for BM.

## Discussion

In this systematic review, we aimed to assess the benefits of RT on the heart of rats and mice fed HFD. We observed that RT positively affects anthropometric, metabolic, functional, and structural parameters altered by HFD.

Some studies have demonstrated that HFD treatment impairs cardiac function and structure, in addition to increasing inflammation and oxidative stress.^9,10,17,18^Although in the study by de Souza Lino et al.^28^ HFD feeding (20%) for 11 weeks did not affect cardiac lipid peroxidation and antioxidant enzymatic activity (e.g., SOD, Mn-SOD, CAT, and GPx) in rats, intervention with RT proved to exert a cardioprotective function. In this sense, after 8 weeks of RT, the animals exhibited lower BM and adiposity index, higher cardiac Mn-SOD and CAT activities, and lower GSH.^28^ These findings are in line with other studies in which an increase in cardiac antioxidant capacity was observed in rats with renovascular hypertension submitted to strength training.^34^

In this context, Effting et al.,^31^ showed that RT was effective in reducing BM and improving glucose metabolism, in addition to neutralizing the increase in oxidative stress (e.g., increase in DCFH and MDA) and in inflammatory markers (e.g., TNF-α) in the hearts of mice fed HFD (i.e., 59% for 26 weeks). The increase in TNF-α after HFD feeding may be explained by the ability of reactive oxygen species to promote lesions in cardiac tissue, which might have led to changes in immune responses.^32^ It is known that oxidative stress triggers pathological cardiac hypertrophy and impairs the contractile function of cardiomyocytes.^35,36^Based on that, cardiac enzymatic antioxidant mechanisms are essential to restore the redox state and avoid exacerbated accumulation of pro-oxidative agents that contribute to cardiac deterioration.^37^ De Souza Lino et al.^28^showed that RT positively regulated Mn-SOD and CAT. However, the study by Effting et al.^31^ indicated that SOD activity did not change and that of CAT was negatively regulated by RT, suggesting that other mechanisms are related to antioxidation, which demands further investigation.

In the study by Kim et al.,^33^ HFD-treated rats (50%) underwent 12 weeks of RT and exhibited reduction in ER stress markers (e.g., pPERK/PERK) in the LV. However, in the same study, the expression of CHOP, another marker of ER stress, was reduced only in the group that performed aerobic exercise, which was associated with the reduction in BM observed only in this training model, denoting the importance of anthropometric control.^33^Because ER stress is associated with oxidative stress and inflammation and is increased in pathological cardiac hypertrophy and heart failure,^38^ these findings indicate cardioprotection induced by RT. Furthermore, RT enhanced LV mitochondrial biogenesis, as evidenced by the increased expression of Cyto-C, SUD, and PGC-1α. Such findings indicate the beneficial effects of RT, since the increase in mitochondrial biogenesis is associated with reduced oxidative stress and apoptosis.^39^Mitochondrial biogenesis has also conferred myocardial protection in a model of heart failure.^40^

Regarding cardiac structural and extracellular matrix remodeling, in 2 studies,^28,29^ RT increased the activity of MMP-2 in the LV of rats fed HFD. It is known that MMP-2 expressed in cardiomyocytes acts directly on the turnover of the extracellular matrix, promoting the degradation of components such as collagen and fibronectin.^41^ Guzzoni et al.^42^ demonstrated that 12 weeks of RT increased the activity of the active MMP-2 isoform, with consequent reduction of its endogenous TIMP-1 inhibitor, which directly contributed to the neutralization of the increase in collagen and fibrosis in the LV in response to aging. As several studies indicate that HFD increases collagen levels and cardiac fibrosis,^33^the upregulation of MMP-2 isoforms in response to RT is important to mitigating damages to the functional properties of the heart.

In most of these studies, the HFD and TR did not directly affect the cardiac biometric properties, which are indirect markers of cardiac hypertrophy. For example, heart and LV masses,^28,29,31,33^ as well as their ratios to tibial length^33^remained unchanged. Only in the study by Leite et al.,^29^ RT increased heart to BM ratio in the group treated with HFD, which decreased in response to RT. Another study with healthy rats also showed that RT increases the LV to BM ratio.^30^

It is well understood that, in the long-term, RT promotes physiological concentric cardiac hypertrophy, characterized by the addition of sarcomeres in parallel, increased cardiac mass, and increased thickness of the LV wall.^43^ Such adaptations improved cardiac contractile function at the organ and cellular levels, without the presence of deleterious changes (i.e., increase in fibrotic tissue, oxidative stress, apoptosis, and inflammation).^43^On the other hand, in the included studies, RT did not alter the heart and LV mass,^28,29,31,33^ or the cross-sectional area and collagen content of LV^33^ in animals fed HFD and commercial chow diet. Based on that, further studies evaluating histomorphometric changes and the signaling pathways involved in cardiac structural remodeling are needed to explore the effects of RT in this model.

Concerning cardiomyocyte function, Melo et al.^32^demonstrated that RT improved the contractile function of cardiomyocytes in rats fed commercial chow diet. Although HFD (49.2% fat for 26 weeks) did not negatively affect cardiomyocyte contractile function (e.g., shortening fraction, contraction and relaxation velocities), the beneficial effect of RT was also not observed in these animals. These findings suggest that HFD-fed rats exhibit resistance to the beneficial effects of RT. Other studies have found that RT improved contractile function in cardiomyocytes in rats with cardiovascular disease.^25^In addition, Lavorato et al.^44^reported that 8 weeks of running on a treadmill increased the contractile function and transient intracellular calcium in cardiomyocytes, whereas HFD (53%) impaired these parameters. More studies are needed to explore the effect of RT on cardiomyocyte function in animals treated with HFD.

Finally, this review has some limitations. First, few studies have examined the effects of TR on HFD; therefore, more studies in this area should be conducted to understand cardiac cellular and molecular mechanisms. Second, the duration of feeding and the percentage of fat in HFD were different between studies, which makes it difficult to homogeneously observe the effects of HFD treatment. For example, the study by Melo et al.^32^ included in the systematic review demonstrated that 26 weeks of HFD (i.e., 49.2%) treatment did not change the cross-sectional area of cardiomyocytes. However, the opposite result was demonstrated in another study that used a diet with a higher percentage of fat (i.e., 60%) for 20 weeks.^45^

In this sense, it is important to highlight the fact that studies with different species (i.e., rat and mouse) and strains (i.e., Wistar, Sprague-Dawley, and Swiss) of rodents were included, which may influence the general results of the HFD effects. Gong et al.^46^ revealed that mice fed HFD (i.e., 45% kcal fat for 16 weeks) showed cardiac hypertrophy at organ (i.e., heart and LV mass) and cellular (i.e., increased cross-sectional area) levels, which was not observed in the included studies. Moreover, the same study by Gong et al.^46^ reported impairments in cardiac contractile function (i.e., fraction of shortening and prolonged time to 90% of relaxation), unlike the findings of Melo et al.^32^ Furthermore, only one study evaluated results related to cardiomyocyte function and cardiac histomorphometry, mitochondrial biogenesis, and ER stress, which require further investigations.

Despite that, our study reveals that the TR and HFD models have not been extensively explored, and such gaps contribute to the direction of future studies on cardiac pathophysiology. In this context, it is important to point out that the percentage of fat present in the HFD and the exposure time are key factors to be considered to clarify the real effects of the diet on cardiac tissue, which makes it possible to compare different interventions in a strongly established model.

Furthermore, we suggest that future studies involving the practice of RT should be explored with different training loads (i.e., intensity, volume, density, and weekly frequency) to determine which model appears to be most appropriate for patients. Moreover, it is extremely important for subsequent studies to evaluate the outcomes of interest presented in the studies included in this systematic review.

## Conclusion

Our results indicate that RT partially counteracts the HFD-induced adverse cardiac remodeling by increasing the activity of structural remodeling markers; elevating mitochondrial biogenesis; reducing oxidative stress, inflammatory marker, and ER stress; and improving hemodynamic, anthropometric, and metabolic parameters.
